# ECMO in resuscitated drowning patients: a propensity score matched sub-analysis—a response to Jouffroy et al.

**DOI:** 10.1186/s13054-023-04705-1

**Published:** 2023-10-27

**Authors:** Thomas Jasny, Jan Kloka, Oliver Old, Florian Piekarski, Gösta Lotz, Kai Zacharowski, Benjamin Friedrichson

**Affiliations:** Department of Anaesthesiology, Intensive Care Medicine and Pain Therapy, University Hospital Frankfurt, Goethe University Frankfurt, Theodor-Stern Kai 7, 60590 Frankfurt, Germany

To the Editor,

We express our gratitude to Romain Jouffroy and Benoît Vivien for their valuable commentary on our manuscript titled "Results from 237 extracorporeal membrane oxygenation runs with drowned patients: a nationwide retrospective study" [1, 2]. These colleagues suggest an analysis of drowned patients managed solely by cardiopulmonary resuscitation (CPR) alone versus those with the combination of CPR and extracorporeal membrane oxygenation (ECMO). Needless to say, that this approach can be an important and insightful sub-analysis of our data set.

The primary objective of our manuscript was to examine the variance among all hospitalized drowning cases, focusing on the comparison of outcomes between those treated with ECMO and those without. Pursuant to this objective, and in light of the sub-analysis proposed by Jouffroy et al., we examined all patients subjected to in-hospital (OPS 8–77*) and out-of-hospital (ICD U69.13!) CPR following drowning events between 2018 and 2020. Due to a change in coding for in-hospital resuscitation and out-of-hospital resuscitation, this short timeframe had to be selected. Using propensity score matching based on age and Elixhauser Score, each patient of the ECMO group was matched with a patient of the non-ECMO group with similar characteristics (Table 1), hence of 122 drowned patients 50% (*n* = 61) received CPR only and 50% (61) received CPR and ECMO [3]. Mortality was 86.9% (*n* = 53) for the ECMO group and 55.7% (*n* = 34) for the non-ECMO group. Logistic regression analysis showed a significant association of increased hospital mortality with V-A ECMO (OR 22.907, CI: 3.652–143.695) and a protective effect for hypertension (OR 0.086, CI: 0.013–0.59) (Fig. 1). Notably, this sub-analysis demonstrated a significantly elevated mortality in the ECMO group compared to the non-ECMO patients. Both groups, after propensity score matching, showed no significant difference in disease severity, measured via the Elixhauser Score (ECMO *n* = 13, IQR: 8–19; Non-ECMO *n* = 11, IQR: 5–20; *p* = 0.66). The potential differences that could have been revealed by clinically established scores, such as SOFA or SAPS II, remain a limitation of our data set [4, 5].

**Table 1:** Patient characteristics for resuscitated drowned patients

	ECMO			Non-ECMO			*p* value
	*N*	%		*N*	%		
Total	61			61			
VV-ECMO	14	23.0					
VA-ECMO	47	77.0					
Death	53	86.9		34	55.7		< 0.0001
Female	19	31.1		16	26.2		0.5482

	Q1	Median	Q3	Q1	Median	Q3	*p* value
Age (year)	16.0	30.0	54.0	18.0	33.0	59.0	0.3937
Hospital stay (h)	7.0	42.6	176.6	16.0	70.5	289.8	0.1773
Elixhauser score	8.0	13.0	19.0	5.0	11.0	20.0	0.6554

Comorbidities	*N*	%		*N*	%		*p* value
Congestive heart failure	11	18.0		11	18.0		1.0000
Hypertension	4	6.6		13	21.3		0.0186
Chronic pulmonary disease	*	*		*	*		
Diabetes	*	*		*	*		
Renal failure	*	*		*	*		
Obesity	0	0.0		3	4.9		0.0795

Complications	*N*	%		*N*	%		*p* value
Intracranial Bleeding	3	4.9		0	0.0		0.0795
Stroke	*	*		*	*		
Pulmonary Embolism	*	*		*	*		
Arterial embolism and/or thrombosis	*	*		*	*		
Myocardial Infarction	*	*		*	*		
CPR prior to admission	49	80.3		54	88.5		0.2119
In-hospital CPR	30	49.2		18	29.5		0.0262
Dialysis	15	24.6		11	18.0		0.3765

*V-V* venovenous, *V-A* venoarterial, ECMO—extracorporeal membrane oxygenation, *CPR* cardiopulmonary resuscitation

*censored < 3 patients

**Fig. 1:**
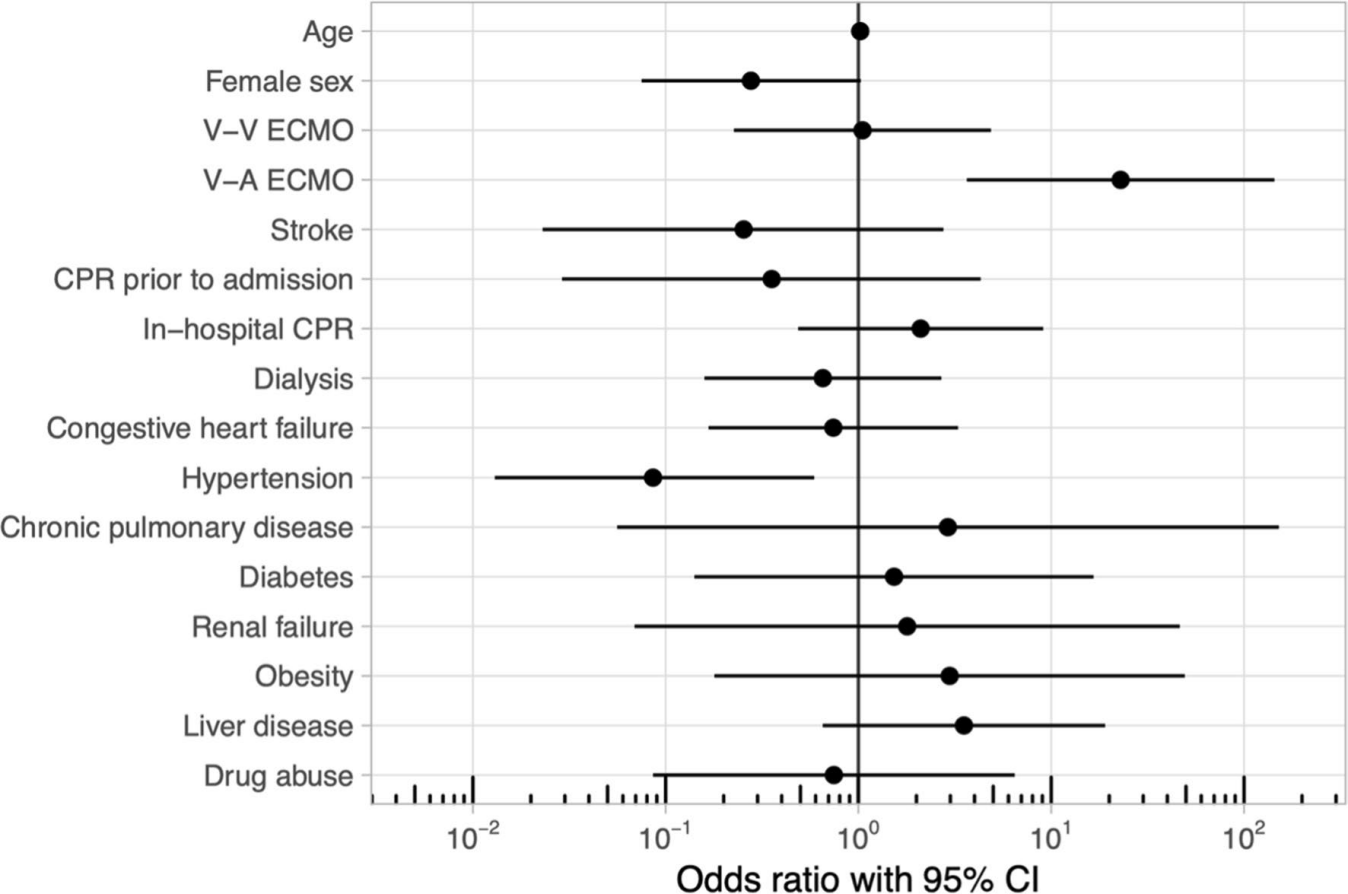
Odds ratio for resuscitated drowned patients with mortality as outcome. V-V: venovenous; V-A: venoarterial; ECMO: extracorporeal membrane oxygenation; CPR: cardiopulmonary resuscitation

To address another important point made by Jouffroy et al. regarding the use of out-of-hospital cardiac arrest (OHCA) predictors, we acknowledge that the database's constraints limit further analysis [2, 6]. It is indeed surprising, both clinically and in the context of the literature, how a better survival with a stroke is associated in drowned patients. Smaller sample size may contribute, and outcomes might be a product of the sample at hand, potentially reversing in other scenarios and relying on secondary data can introduce inherent biases. Strokes might be under-diagnosed in non-survivors due to early fatalities, whereas survivors receive more comprehensive diagnostics. However, our sub-analysis of resuscitated drowned patients did not reproduce this finding.

Regarding the comment about potential censors in the multivariate logistic regression, such as chronic pulmonary disease, it is imperative to clarify that our logistic model, which was processed on the servers of the Federal Statistical Office, contained uncensored data. The censorship of our published results is solely for privacy and data protection reasons, and therefore, a censored value may be 1 or 2.

In conclusion, our sub-analysis vividly illustrates an extremely high mortality rate of more than 86% for resuscitated patients on ECMO, with a significantly increased in-hospital mortality associated with V-A ECMO. As previously emphasized, further studies are urgently required to evaluate the tangible benefits of ECMO therapy for resuscitated drowning victims.

## Data Availability

The Federal Statistical Office of Germany provided all data used. According to §21KHEntgG, reimbursement data are free for scientific use, but its availability is restricted and not publicly available. Upon reasonable request and with permission of the Federal Statistical Office of Germany, data are available from the authors.
